# A COVID-19 Epidemic Model Predicting the Effectiveness of Vaccination in the US

**DOI:** 10.3390/idr13030062

**Published:** 2021-07-26

**Authors:** Glenn Webb

**Affiliations:** Department of Mathematics, Vanderbilt University, Nashville, TN 37240, USA; glenn.f.webb@vanderbilt.edu

**Keywords:** COVID-19, data, transmission, asymptomatic, symptomatic, vaccination

## Abstract

A model of a COVID-19 epidemic is used to predict the effectiveness of vaccination in the US. The model incorporates key features of COVID-19 epidemics: asymptomatic and symptomatic infectiousness, reported and unreported cases data, and social measures implemented to decrease infection transmission. The model analyzes the effectiveness of vaccination in terms of vaccination efficiency, vaccination scheduling, and relaxation of social measures that decrease disease transmission. The model demonstrates that the subsiding of the epidemic as vaccination is implemented depends critically on the scale of relaxation of social measures that reduce disease transmission.

## 1. Introduction

The objective of this study is to predict the outcome of vaccine implementation for the mitigation of the COVID-19 epidemic in the United States. Vaccine distribution began in the US on 14 December 2020. As of 15 June 2021, approximately 148,000,000 people have been fully vaccinated, approximately 45% of the total US population. (https://covid.cdc.gov/covid-data-tracker/#vaccination-demographic (accessed on 15 June 2021)). Vaccination offers great hope for curtailment and elimination of the COVID-19 pandemic, but there is uncertainty in terms of vaccine effectiveness, vaccine opposition, and the consequences of resumption of normal social behaviour as the number of vaccinated people increases. This study addresses these issues with a mathematical model incorporating key features of COVID-19 epidemics and key features of COVID-19 vaccination implementation. In our References we have listed relevant studies of mathematical models of COVID-19 vaccination implementation [1,2,3,4,5,6,7,8,9,10,11,12,13,14,15,16,17,18,19,20,21,22,23,24,25,26,27,28,29,30,31,32,33,34,35,36,37,38,39,40,41,42,43,44,45,46,47,48,49,50,51,52,53,54,55,56,57,58,59,60,61,62,63,64,65,66,67,68,69,70,71,72,73,74,75,76,77,78,79,80,81]. Our study brings new understanding of vaccination implementation to COVID-19 models, in our focus on the existing daily reported cases to parameterize the model and extend the model time-frame outcomes to varied vaccine efficiencies and varied social behavior restoration.

A key issue in developing a mathematical model of a COVID-19 epidemic, is informing the model dynamics in terms of reported epidemic data. For the US, this data consists of daily reported cases to the Centers for Disease Control and Prevention (CDC). In the US, daily cases are reported by jurisdictional health departments through the National Notifiable Diseases Surveillance System (NNDSS), as well as through resources provided by the CDC COVID-19 response (https://www.cdc.gov/coronavirus/2019-ncov/hcp/clinical-guidance-management-patients.html (accessed on 16 February 2021)).

This daily reported cases data is extremely erratic, and subject to on-going updating. A standard method of managing this data is to use a rolling weekly averaging of the daily reported values. The rolling weekly averaged data still fluctuates considerably, which makes the dynamic infection transmission parameters of the model difficult to establish. In this study, the formulation of the model will be used to identify the transmission parameterisation of the model in terms of the rolling weekly average daily reported cases. For the projection forward in time after the last date of daily reported cases, the infection transmission parameters will be extrapolated from the most recent daily reported data.

Another key issue in developing a mathematical model of a COVID-19 epidemic concerns the fraction of reported cases and fraction of unreported cases. These fractions are critical in estimating the number of people still susceptible to infection as vaccination implementation proceeds, since a sizeable number of people vaccinated have already been infected and have significant immunity to re-infection [78]. As of 14 April 2021, the CDC estimated that 1 in 4.3 COVID-19 infections were reported and 1 in 3.9 symptomatic illnesses were reported. (https://www.cdc.gov/coronavirus/2019-ncov/cases-updates/burden.html (accessed on 16 February 2021)) In this study, it will be assumed that 1 in 4 of total COVID-19 cases have been reported in the US.

Other key issues in COVID-19 model development concern the lengths of the asymptomatic infectiousness period and the symptomatic infectiousness periods for reported and unreported cases [10,11,39,43]. Since people who are asymptomatic are not always tested, the prevalence of asymptomatic infection and detection of pre-symptomatic infection is not yet well understood (https://www.cdc.gov/coronavirus/2019-ncov/hcp/clinical-guidance-management-patients.html (accessed on 16 February 2021)). All these issues concerning parameterisation relate to the impact of social distancing measures that reduce disease transmission. As vaccination proceeds, there is a reduction of these social distancing measures, which effects the fraction of people not susceptible to infection, so-called herd immunity. In this study these issues will be examined for projections for the subsiding of the COVID-19 epidemic in the US as vaccination proceeds. In this study, the COVID-19 model projections for the US will show that epidemic will subside to low levels in late 2021 and early 2022.

In an earlier work, ref. [79] a method similar to the method developed here was used to evaluate the COVID-19 vaccination program in the United Kingdom. In [79], it was shown that the COVID-19 vaccination program in the UK would cause the epidemic to subside to a very low level by early 2022. In future works, these methods will be applied to other countries and locations. These future works will utilise the many studies of mathematical models of COVID-19 epidemics listed in the References.

## 2. Materials and Methods

**Figure 1:**
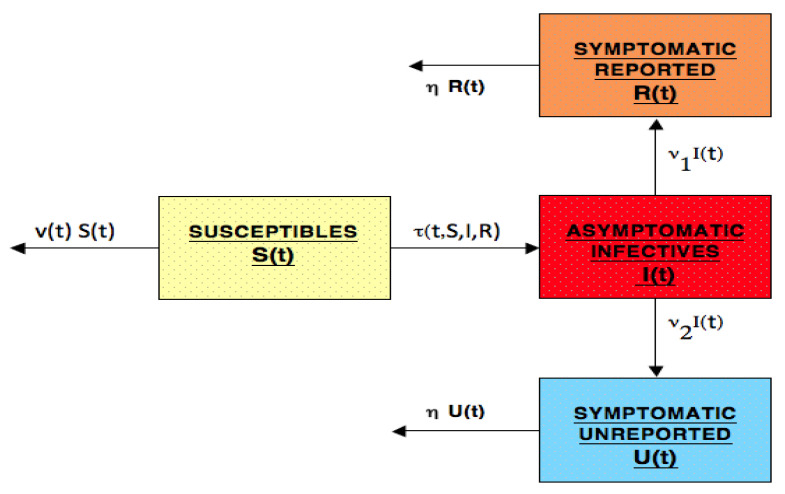
Flow diagram of the model compartments: susceptible, asymptomatic infected, reported symptomatic infected, and unreported symptomatic infected. The time units are days.

The model is a system of ordinary differential equations for the epidemic population compartments. The compartments are S(t) = susceptible individuals at time *t*, I(t) = asymptomatic infectious individuals at time *t*, R(t) = symptomatic infectious individuals at time *t* who will be reported, and U(t) = symptomatic infectious individuals at time *t* who will not be reported, The flow diagram of the model is shown in Figure 1. The equations of the model are as follows:

(1) $$\begin{array}{ccc} S^{\prime }\left(t\right) & = & -\tau \left(t,S\left(t\right),I\left(t\right),R\left(t\right)\right)\;-v\left(t\right)S\left(t\right),\;t\geq t_{0}, \end{array}$$

(2) $$\begin{array}{ccc} I^{\prime }\left(t\right) & = & \tau \left(t,S\left(t\right),I\left(t\right),R\left(t\right)\right)-\left(\nu_{1}+\nu_{2}\right)I\left(t\right),\;t\geq t_{0}, \end{array}$$

(3) $$\begin{array}{ccc} R^{\prime }\left(t\right) & = & \nu_{1}I\left(t\right)-\eta R\left(t\right),\;t\geq t_{0}, \end{array}$$

(4) $$\begin{array}{ccc} U^{\prime }\left(t\right) & = & \nu_{2}I\left(t\right)-\eta U\left(t\right),\;t\geq t_{0}. \end{array}$$

The model parameters of the COVID-19 epidemic in the US are given below.

### 2.1. The Transmission Rate before the Last Day of Daily Reported Cases

**Figure 2:**
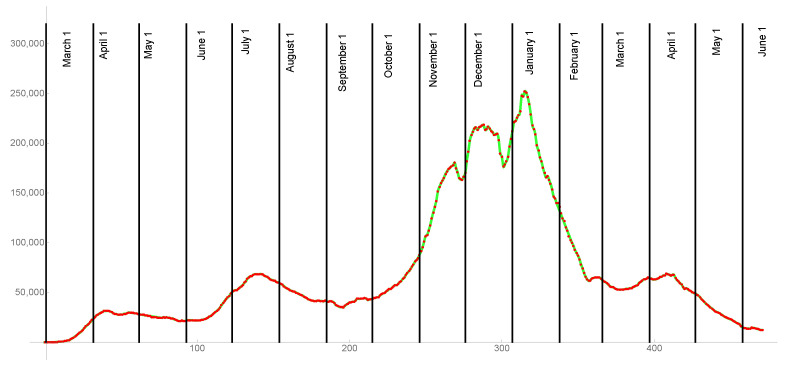
Red dots are discrete rolling weekly averaged daily reported cases from 1 March 2020 to 15 June 2021, and the green graph is the continuum cubic spline interpolation CS(t) of the red dots.

The time-dependent transmission rate in the model before the last date of daily reported cases, is obtained from the daily reported cases data. A reported case is defined by clinical or laboratory criteria (https://ndc.services.cdc.gov/conditions/coronavirus-disease-2019-covid-19/ (accessed on 15 June 2021)). Since the daily reported cases data is typically very erratic, a rolling weekly average of the daily reported cases data is used to smooth this data. The discrete smoothing of the daily reported cases data to rolling weekly average values, can be interpolated by a continuum cubic spline curve CS(t). This curve is constructed by defining cubic polynomials on successive pairs of intervals $\left[t_{1},\;t_{2}\right],\;\left[t_{2},\;t_{3}\right],\;\left[t_{3},\;t_{4}\right],\;\left[t_{4},\;t_{5}\right],\ldots$, where the interpolation agrees with the rolling weekly average daily cases data at the integer values, and is three times differentiable from the first to last day of rolling weekly average daily cases. In Figure 2 the graphs of the daily reported cases data, the rolling weekly averaged daily reported cases data, and the cubic spline interpolation of the rolling weekly averaged daily reported cases data are shown for the US from 1 March 2020 to 15 June 2021.

Let $dr\left(t_{1}\right),\;dr\left(t_{2}\right),\ldots$ be the rolling weekly average number of daily reported cases each day, from the first week of March, 2020 up to the last day of daily reported cases 15 June 2021, where time $t_{1},\;t_{2},\ldots$ is discrete, day by day. In the model, the continuum version DR(t) of $dr\left(t_{1}\right),\;dr\left(t_{2}\right),\ldots$, can be assumed to satisfy

(5) $$DR^{\prime }\left(t\right)=\nu_{1}\;I\left(t\right)-DR\left(t\right)\;\Rightarrow \;I\left(t\right)=\left(\;\frac{DR^{\prime }\left(t\right)+DR\left(t\right)}{\nu_{1}}\;\right).$$

Model Equation (2) implies the transmission rate τ(t, S(t), I(t), R(t)) satisfies, until the last day of reported cases data,

$$\begin{array}{ccc} \tau (t,S(t),I(t),R(t)) & = & I^{\prime }\left(t\right)+\left(\nu_{1}+\nu_{2}\right)\;I\left(t\right)\\ & = & \frac{DR^{\prime \prime }\left(t\right)+DR^{\prime }\left(t\right)}{\nu_{1}}\;+\;\left(\nu_{1}+\nu_{2}\right)\;\left(\;\frac{DR^{\prime }\left(t\right)+DR\left(t\right)}{\nu_{1}}\;\right). \end{array}$$

DR(t) in (5) for the model can be equated to the continuum cubic spline interpolation CS(t) of the discrete rolling weekly averaged data, and the derivatives $DR^{\prime }\left(t\right)=CS^{\prime }\left(t\right)$ and $DR^{\prime \prime }\left(t\right)=CS^{\prime \prime }\left(t\right)$ can also be obtained. Thus, the continuum interpolation CS(t) derived from the rolling weekly average daily data agrees exactly with this data at discrete day by day values, and has continuous first and second derivatives on its domain. The continuum time-dependent transmission rate in the model before the last date of daily reported cases, is thus given by

(6) $$\tau \left(t,S\left(t\right),I\left(t\right),R\left(t\right)\right)=\frac{CS^{\prime \prime }\left(t\right)+CS^{\prime }\left(t\right)}{\nu_{1}}\;+\;\left(\nu_{1}+\nu_{2}\right)\;\left(\;\frac{CS^{\prime }\left(t\right)+CS\left(t\right)}{\nu_{1}}\;\right).$$

**Figure 3:**
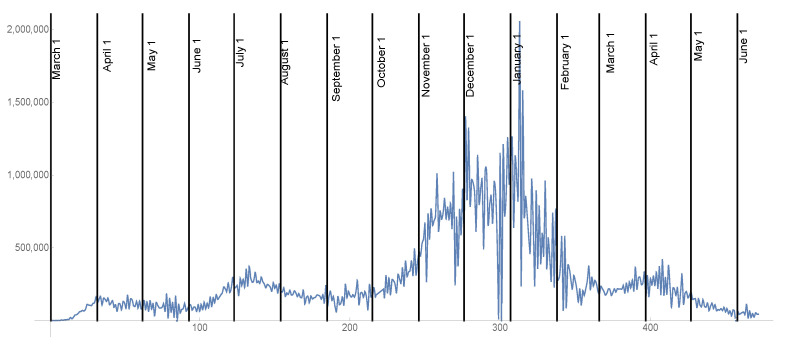
The transmission rate before the last date of reported daily cases, as in (6) for the COVID-19 epidemic in the US from 7 March 2020 to 15 June 2021.

In Figure 3, the transmission rate as in (6), is graphed from 7 March 2020 to 15 June 2021.

The model with this form for the transmission dynamics provides information about S(t),I(t),R(t), and U(t) up to the last date of daily reported cases. This method to parameterize the transmission rate using daily reported cases data was used in [79]. Similar methods have been used in [18,27,28,46,47,48] to relate reported cases data to model dynamics.

### 2.2. The Transmission Rate after the Last Day of Daily Reported Cases

After the last day of daily reported cases, the transmission dynamics can be extrapolated, based on their most recent history before this last date, and the dynamics of the epidemic can be projected forward in time. After the last day of daily reported cases, the transmission rate has the standard mass-action form $\hat{\tau }\left(t\right)\;\left(I\left(t\right)+4R\left(t\right)\right)\;S\left(t\right))$, where it is assumed that asymptomatic cases, unreported symptomatic cases, and reported symptomatic cases have equal likelihood of transmission to susceptibles. The ratio of unreported symptomatic cases and reported symptomatic cases is assumed to be 3 to 1. The function $\hat{\tau }\left(t\right)$ incorporates the transmission rate before the last day of daily reported cases, as well as the time dependent relaxation of social distancing behavior as vaccination is implemented.

Before the last day 15 June 2021, of daily reported cases, the transmission rate in (1) has the form as in (6). After time $t_{D}=$ 15 June 2021, a time $t_{1}=$ 1 July 2021 is set such that there is an increasing return to normalcy of social distancing behaviour, and the transmission rate in (1) has the form for $t_{D}\leq \;t\leq \;t_{1}$

$$\tau \left(t,S\left(t\right),I\left(t\right),R\left(t\right)\right)=(\tau (t_{D},S\left(t_{D}\right),I\left(t_{D}\right),R\left(t_{D}\right)))\left(\frac{\left(I(t)+4R(t))S(t\right)}{\left(I\left(t_{D}\right)+4R\left(t_{D}\right)\right)S\left(t_{D}\right)}\right).$$

After time $t_{1}=$ 1 July 2021, a later time $t_{2}=$ 1 October 2021 is set, such that there is a further return to normalcy of social distancing behaviour, involving a scaling factor ω. For $t_{1}\leq \;t\;\leq t_{2}$, the transmission rate has the form

τ(t,S(t),I(t),R(t))=

$$(1.0+\omega (t-t_{1}))(\tau (t_{D}),S\left(t_{D}\right),I\left(t_{D}\right),R\left(t_{D}\right)))(\frac{\left(I(t)+4R(t))S(t\right)}{\left(I\left(t_{D}\right)+4R\left(t_{D}\right)\right)S\left(t_{D}\right)}).$$

After time $t_{2}=$ 1 October 2021, there is no further change in social distancing behaviour. For $t_{2}\leq \;t$, the transmission rate has the form

τ(t,S(t),I(t),R(t))=

$$(1.0+\omega (t_{2}-t_{1}))(\tau (t_{D},S\left(t_{D}\right),I\left(t_{D}\right),R\left(t_{D}\right)))(\frac{\left(I(t)+4R(t))S(t\right)}{\left(I\left(t_{D}\right)+4R\left(t_{D}\right)\right)S\left(t_{D}\right)}).$$

The transmission rate is continuous, and in particular, continuous at day $t_{D}=$ 15 June 2021, day $t_{1}=$ 1 July 2021, and day $t_{2}=$ 1 October 2021. The magnitude of the parameter ω, corresponding to level of resumption of normal social distancing behaviour, is critical for resurgence of the epidemic.

The formulas for the transmission rates after the last day of daily reported cases can be interpreted as corresponding to a emergence of a new viral strain with greater transmissibility, as well as a restoration of normal social behavior. Vaccination could be less efficient for the new viral strain, and result in greater transmissibility, with dynamics represented by these formulas.

### 2.3. The Rates of Transition from Asymptomatic Infection to Symptomatic Infection

Asymptomatic infectious individuals I(t) are infectious for an average period of one week before being symptomatic. The fraction 1/4 of asymptomatic infectious become symptomatic R(t) at rate $\nu_{1}=0.25/7$ per day, and the fraction 3/4 become unreported symptomatic infectious at rate $\nu_{2}=0.75/7$ per day. Reported symptomatic individuals have transmission capability for an average period of one week before becoming incapable of transmission, and the same average period of transmissibility holds for unreported symptomatic individuals. Thus, η=1.0/7 days. The values for $\nu_{1}$, $\nu_{2}$, and η are assumed, and consistent with current information about transmissibility of COVID-19 infection (https://www.cdc.gov/coronavirus/2019-ncov/hcp/faq.html (accessed on 15 June 2021)).

### 2.4. The Rate of Vaccination

**Figure 4:**
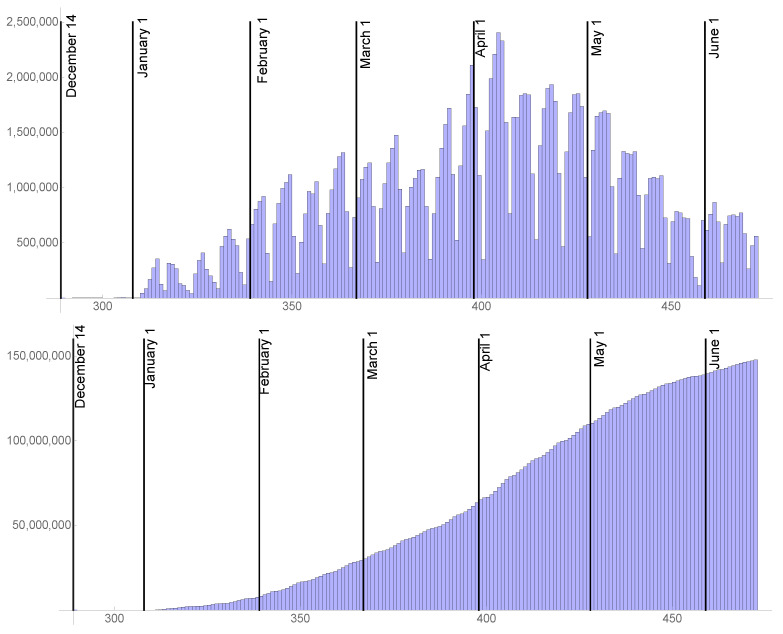
Daily vaccination data $v_{daily}\left(t\right)$ (**top**) and cumulative version CV(t) of this data (**bottom**) for the US from 14 December 2020 to 15 June 2021, as step functions in continuous time. After 15 June 2021, $v_{data}\left(t\right)$ = 1,000,000 per day is assumed constant until $t=t_{Vmax}$. After $t_{Vmax}$, it is 0.

In (1), susceptible individuals are removed from the possibility of infection at a rate v(t) per day, as a result of vaccination, where this time dependent rate assumes they are fully vaccinated. In Figure 4, the daily number of vaccinated individuals $v_{daily}\left(t\right)$ and cumulative version of $v_{daily}\left(t\right)$ are graphed from 14 December 2020 to 15 June 2021 (https://covid.cdc.gov/covid-data-tracker/vaccination-trends (accessed on June 15 2021)). After the last day of daily reported vaccination data 15 June 2021, $v_{daily}\left(t\right)$ is assumed to be constant at 1,000,000 per day until a later date $t_{Vmax}$. After $t_{Vmax}$, the daily vaccination rate $v_{daily}\left(t\right)=0$. The date $t_{Vmax}$ will be set to values that represent the ultimate fraction of the US population vaccinated at 90%, 85%, and 80% of the total population. These fractions incorporate vaccination resistance within the US population. In (1), $v\left(t\right)=0.95\;v_{daily}\left(t\right)\;S\left(t\right)/\left(S\left(0\right)-CV\left(t\right)\right)$, where CV(t)= the cumulative number of vaccinated individuals up to time *t*. The efficiency of vaccination is assumed to be 95%, and the removal of susceptibles due to vaccination is the fraction S(t)/(S(0)−CV(t)) of the number vaccinated, which excludes individuals vaccinated who were previously infected. The future daily vaccination rates are chosen for illustration, since their values are very uncertain.

## 3. Results

**Figure 5:**
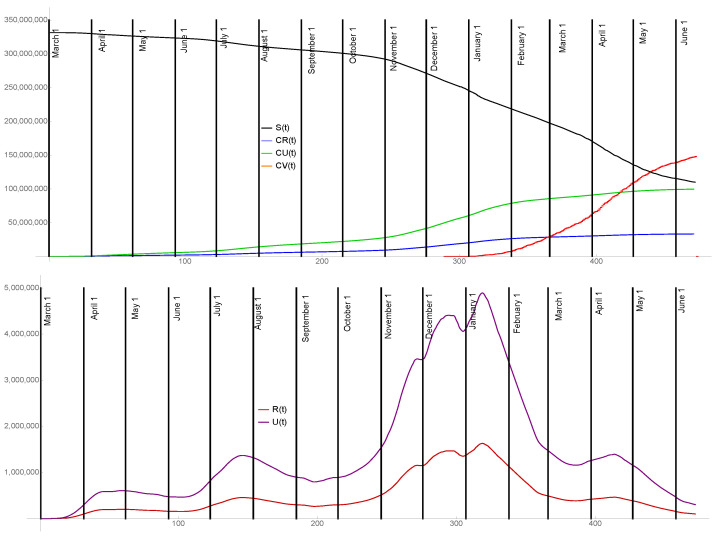
Model output from 7 March 2020 to the last day of daily reported cases data 15 June 2021. The graphs are S(t) (black), R(t) (magenta), U(t) (purple), CR(t) (blue), CU(t) (green), and CV(t) (orange).

Set $t_{0}=7=$ 7 March 2020. Set $S(t_{0})$ = 331,500,000, the population of the US according to the April 2020 census. Set $I(t_{0})=1$, $R(t_{0})=1$, $U(t_{0})=1$. The model output before the last day of daily reported cases 15 June 2021, is graphed in Figure 5. For $t_{D}=\;$15 June 2021, $S(t_{D})$ = 109,931,000, $I(t_{D})$ = 339,353, $R(t_{D})$ = 101,039, $U(t_{D})$ = 303,117, and $\tau (t_{D},S\left(t_{D}\right),I\left(t_{D}\right),R\left(t_{D}\right))$ = 44,188. The graphs of the model compartments S(t),R(t),U(t), the cumulative reported cases CR(t), the cumulative unreported cases CU(t), and the cumulative vaccinated individuals CV(t) are shown. The cumulative unreported cases CU(t) and cumulative reported cases CR(t) are in a ratio of 3 to 1, as are the unreported cases U(t) and reported cases R(t).

After 15 June 2021, the last day of daily reported cases, the model is projected forward to 1 January 2022, to predict outcomes of vaccination implementation for varying scenarios involving the fraction of the population vaccinated and the level of return to normal social distancing behaviour. The ultimate fraction of the population vaccinated is set to 90%, 85% and 80% of the total population. The restoration of normal social behaviour as vaccination proceeds is scaled to three different levels, as determined by ω. These outcomes, all together, predict the effect of vaccination for the COVID-19 epidemic in the US.

### 3.1. 90% of the Population Becomes Fully Vaccinated

After the last day $t_{D}=$ 15 June 2021, of daily reported cases, for 90% of the population to be ultimately vaccinated, the daily vaccination rate $v_{daily}\left(t\right)$ is

$$\begin{array}{ccc} v_{daily}\left(t\right) & = & 1,000,000,\;15\;\mathrm{June}\;2021\;\leq t\;\leq t_{Vmax}\;=\;11\;\mathrm{November}\;2021\\ v_{daily}\left(t\right) & = & 0,\;t_{Vmax}=\;11\;\mathrm{November}\;2021\;<\;t. \end{array}$$

**Figure 6:**
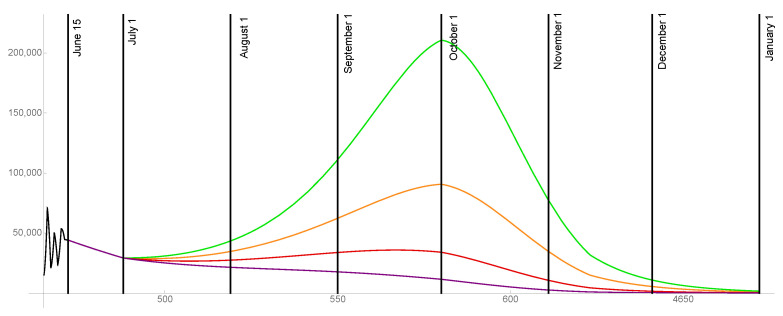
Transmission rates for the level of social distancing resumption ω=0.03 (green), ω=0.025 (orange), ω=0.02 (red), ω=0.015 (purple). The ultimate vaccinated population reaches 90%.

**Figure 7:**
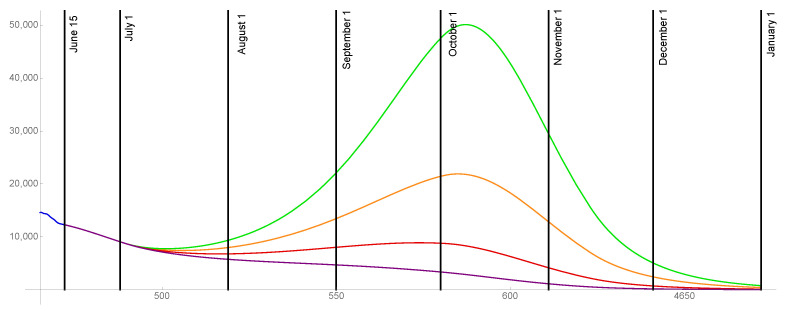
Model outcomes for the daily reported cases DR(t), for the level of social distancing resumption ω=0.03 (green), ω=0.025 (orange), ω=0.02 (red), ω=0.015 (purple).

For the case that 90% of the population is ultimately vaccinated, the transmission rates are graphed in Figure 6 and the daily reported cases are graphed in Figure 7 for ω=0.015,0.02,0.025,0.03.

### 3.2. 85% of the Population Becomes Fully Vaccinated

After the last day $t_{D}=$ 15 June 2021, of daily reported cases, for 85% of the population to be ultimately vaccinated, the daily vaccination rate $v_{daily}\left(t\right)$ is

$$\begin{array}{ccc} v_{daily}\left(t\right) & = & 1,000,000,\;15\;\mathrm{June}\;2021\;\leq t\;\leq t_{Vmax}\;=\;26\;\mathrm{October}\;2021\\ v_{daily}\left(t\right) & = & 0,\;t_{Vmax}=\;26\;\mathrm{October}\;2021\;<\;t. \end{array}$$

**Figure 8:**
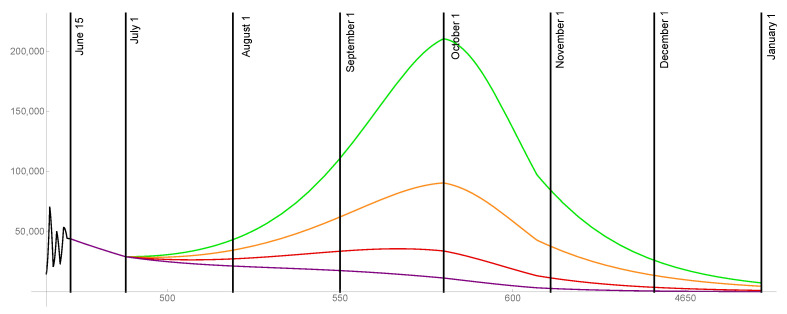
Transmission rates for the level of social distancing resumption ω=0.03 (green), ω=0.025 (orange), ω=0.02 (red), ω=0.015 (purple). The ultimate vaccinated population reaches 85%.

**Figure 9:**
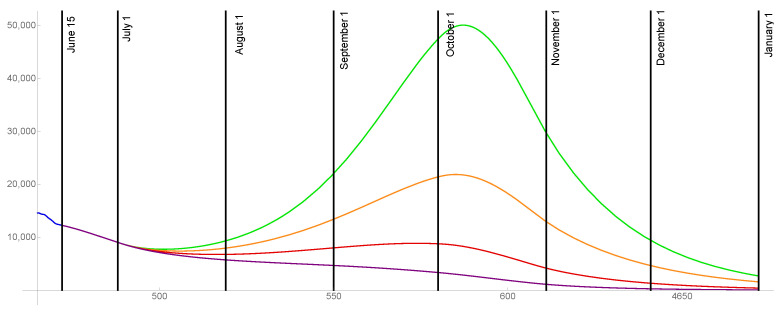
Model outcomes for the daily reported cases DR(t), for the level of social distancing resumption ω=0.03 (green), ω=0.025 (orange), ω=0.02 (red), ω=0.015 (purple).

For the case that 85% of the population is ultimately vaccinated, the transmission rates are graphed in Figure 8 and the daily reported cases are graphed in Figure 9 for ω=0.015,0.02,0.025,0.03.

### 3.3. 80% of the Population Becomes Fully Vaccinated

After the last day $t_{D}=$ 15 June 2021, of daily reported cases, for 80% of the population to be ultimately vaccinated, the daily vaccination rate $v_{daily}\left(t\right)$ is

$$\begin{array}{ccc} v_{daily}\left(t\right) & = & 1,000,000,\;15\;\mathrm{June}\;2021\;\leq t\;\leq t_{Vmax}\;=\;9\;\mathrm{October}\;2021\\ v_{daily}\left(t\right) & = & 0,\;t_{Vmax}=\;9\;\mathrm{October}\;2021\;<\;t. \end{array}$$

**Figure 10:**
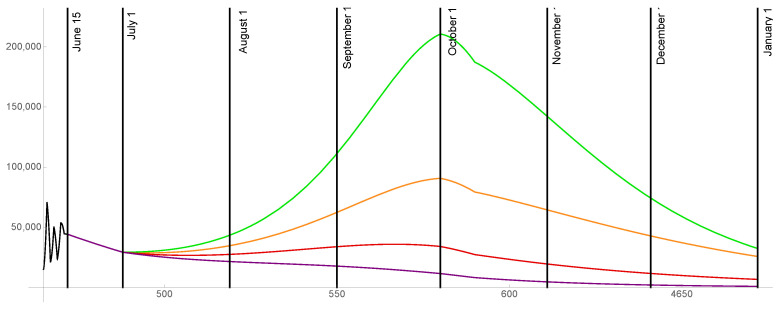
Transmission rates for the level of social distancing resumption ω=0.03 (green), ω=0.025 (orange), ω=0.02 (red), ω=0.015 (purple). The ultimate vaccinated population reaches 80%.

**Figure 11:**
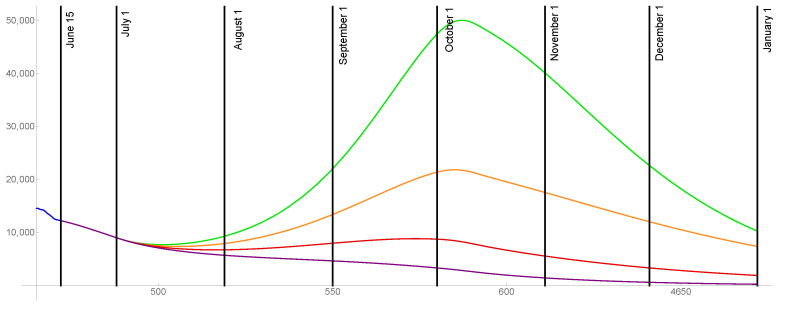
Model outcomes for the daily reported cases DR(t), for the level of social distancing resumption ω=0.03 (green), ω=0.025 (orange), ω=0.02 (red), ω=0.015 (purple).

For the case that 80% of the population is ultimately vaccinated, the transmission rates are graphed in Figure 10 and the daily reported cases are graphed in Figure 11 for ω=0.015,0.02,0.025,0.03.

The model output from Figure 7, Figure 9, and Figure 11 is summarized in Table 1 for time t= 1 January 2022.

## 4. Discussion

A model of a COVID-19 epidemic is used to predict the effectiveness of vaccination in the United States. The model incorporates basic elements of COVID-19 dynamics: transmission due to asymptomatic and symptomatic infected individuals, transmission due to reported and unreported cases, and transmission mitigation due to social distancing measures. A rolling weekly averaging method is used to smooth the highly variable daily cases data reported to the CDC. The model formulation is constructed so that the daily reported cases in the model is in agreement with the rolling weekly averaged daily cases data reported to the CDC.

The model dynamics are projected forward from the last day 15 June 2021 of daily reported cases data, in order to examine the effectiveness of vaccination in controlling the epidemic. The vaccination rate forward from 15 June 2021 is set so that the ultimate number of people vaccinated is 90%, 85%, or 80% of the US population, which varies due to vaccine hesitancy and opposition within the US population [75]. As time proceeds forward from 15 June 2021, the transmission rate is moderated, in correspondence with a restoration of normal social distancing, as the number of susceptible individuals is reduced due to vaccination. Between 15 June 2021 and 1 July 2021, the transmission rate increases due to a relaxation of social distancing behaviour, from the transmission rate before the last date 15 June 2021 of daily reported cases. Between 1 July 2021 and 1 October 2021, the transmission rate increases still further due to a relaxation of social distancing behaviour, according to a scaling factor that corresponds to the level of reduction of social distancing measures. The model is simulated, with these assumptions on the ultimate vaccination level and the reduction of social distancing measures level, as vaccination implementation proceeds.

The model simulations predict the following outcomes of the daily reported cases between 15 June 2021 and 1 January 2022: 

(1) For higher vaccination levels and lower levels of social distancing restoration, the daily reported cases decrease rapidly to very low levels by 1 January 2022. 

(2) For lower vaccination levels and higher levels of social distancing restoration, the daily reported cases first increase slowly, and then decrease to relatively low levels by 1 January 2022. 

The model predicts that the COVID-19 epidemic in the US will not extinguish completely in 2022, but will subside to a level that allows a return to normal social distancing behaviours. The future of the COVID-19 epidemic in the US could be very different, if new more virulent and vaccine-resistant strains develop, and are imported into the US from other countries.

In future works, COVID-19 epidemics in other countries and locations will be examined. Extensions of the model will be developed to address other issues in COVID-19 epidemics, including viral strains, vaccine efficiency, age based, demographic based, geographic based population variation, as well as disease age of infected individuals, and vaccination age of vaccinated individuals in the epidemic populations.

## Figures and Tables

**Table 1:** Model simulations for daily reported cases DR(t), susceptibles S(t), and cumulative reported cases $CR\left(t\right)-CR\left(t_{D}\right)$ since the last date of data, where t= 1 January 2022 for fully vaccinated =90%,85%,80% and social behaviour scaling factor ω=0.03,0.025,0.02,0.015.

Vaccinated	ω=0.03	ω=0.025	ω=0.02	ω=0.015
90%	DR(t)=761	DR(t)=398	DR(t)=105	DR(t)=18
	S(t) = 14,768,000	S(t) = 17,985,000	S(t) = 19,771,000	S(t) = 20,570,000
	$CR\left(t\right)-CR\left(t_{D}\right)=$	$CR\left(t\right)-CR\left(t_{D}\right)=$	$CR\left(t\right)-CR\left(t_{D}\right)=$	$CR\left(t\right)-CR\left(t_{D}\right)=$
	3,768,000	2,068,000	1,155,000	745,000
85%	DR(t)=2651	DR(t)=1558	DR(t)=397	DR(t)=62
	S(t) = 20,971,000	S(t) = 26,023,000	S(t) = 28,821,000	S(t) = 29,954,000
	$CR\left(t\right)-CR\left(t_{D}\right)=$	$CR\left(t\right)-CR\left(t_{D}\right)=$	$CR\left(t\right)-CR\left(t_{D}\right)=$	$CR\left(t\right)-CR\left(t_{D}\right)=$
	3,967,000	2,171,000	1,184,000	750,000
80%	DR(t) = 10,147	DR(t)=7352	DR(t)=1915	DR(t)=265
	S(t) = 25,486,000	S(t) = 33,142,000	S(t) = 37,926,000	S(t) = 34,712,000
	$CR\left(t\right)-CR\left(t_{D}\right)=$	$CR\left(t\right)-CR\left(t_{D}\right)=$	$CR\left(t\right)-CR\left(t_{D}\right)=$	$CR\left(t\right)-CR\left(t_{D}\right)=$
	4,788,000	2,624,000	1,310,000	774,000

## Data Availability

Not applicable.
